# Vilnius Declaration on chronic respiratory diseases: multisectoral care pathways embedding guided self-management, mHealth and air pollution in chronic respiratory diseases

**DOI:** 10.1186/s13601-019-0242-2

**Published:** 2019-01-28

**Authors:** A. Valiulis, J. Bousquet, A. Veryga, U. Suprun, D. Sergeenko, S. Cebotari, D. Borelli, S. Pietikainen, J. Banys, I. Agache, N. E. Billo, A. Bush, I. Chkhaidze, L. Dubey, W. J. Fokkens, J. Grigg, T. Haahtela, K. Julge, O. Katilov, N. Khaltaev, M. Odemyr, S. Palkonen, R. Savli, A. Utkus, V. Vilc, T. Alasevicius, A. Bedbrook, M. Bewick, J. Chorostowska-Wynimko, E. Danila, A. Hadjipanayis, R. Karseladze, V. Kvedariene, E. Lesinskas, L. Münter, B. Samolinski, S. Sargsyan, B. Sitkauskiene, D. Somekh, L. Vaideliene, A. Valiulis, P. W. Hellings

**Affiliations:** 1 Department of Public Health, Clinic of Children’s Diseases, and Institute of Health Sciences, Vilnius University Institute of Clinical Medicine, Vilnius, Lithuania; 2 European Academy of Paediatrics (EAP/UEMS-SP), Brussels, Belgium; 3 MACVIA-France, Fondation partenariale FMC VIA-LR, CHU Montpellier, 371 Avenue du Doyen Gaston Giraud, 34295 Montpellier Cedex 5, France; 4 INSERM U 1168, VIMA : Ageing and Chronic Diseases Epidemiological and Public Health Approaches, Villejuif, France; 5 UMR-S 1168, Université Versailles St-Quentin-en-Yvelines, Montigny le Bretonneux, France; 6 Euforea, Brussels, Belgium; 7 Charité, Berlin, Germany; 8 Minister of Health, Vilnius, Lithuania; 9 Minister of Health, Kiev, Ukraine; 10 Minister of Labour, Health and Social Affairs, Tbilisi, Georgia; 11 Minister of Health, Labour and Social Protection, Chișinău, Moldova; 12 European Parliament, Perugia, Italy; 13 European Parliament, Helsinki, Finland; 14 Lithuianian Academy of Sciences, Vilnius, Lithuania; 15 Faculty of Medicine, Transylvania University, Brasov, Romania; 16 Global Alliance Against Chronic Respiratory Diseases (GARD), Joensuu, Finland; 17 Imperial College and Royal Brompton Hospital, London, UK; 18 Department of Pediatrics, and Iashvili Central Children’s Hospital, Tbilisi State Medical University, Tbilisi, Georgia; 19 Faculty of Postgraduate Education, Lviv National Medical University by Danylo Halytsky, Lviv, Ukraine; 20 Department of Otorhinolaryngology, Amsterdam University Medical Centres, AMC, Amsterdam, The Netherlands; 21 Centre for Genomics and Child Health, Blizard Institute, Queen Mary University of London, London, UK; 22 Skin and Allergy Hospital, Helsinki University Hospital and University of Helsinki, Helsinki, Finland; 23 Children’s Clinic, Tartu University Institute of Clinical Medicine, Tartu, Estonia; 24 Vinnytsa National Medical University by Mykola Pyrogov, Vinnytsa, Ukraine; 25 Global Alliance Against Chronic Respiratory Diseases (GARD-WHO), Geneva, Switzerland; 26 European Federation of Allergy and Airways Diseases Patients’ Associations (EFA), Brussels, Belgium; 27 Department of Human and Medical Genetics, Institute of Biomedical Sciences, Vilnius University Faculty of Medicine, Vilnius, Lithuania; 28 Association of Medical Schools in Europe, Berlin, Germany; 29 State Institute of Phtysiopulmonology by Chiril Draganiuk, Chisinau, Moldova; 30 iQ4U Consultants Ltd, London, UK; 31 Department of Genetics and Clinical Immunology, National Institute of Tuberculosis and Lung Diseases, Warsaw, Poland; 32 Clinic of Chest Diseases, Immunology and Allergology, Centre of Pulmonology and Allergology, Institute of Clinical Medicine, Vilnius University Medical Faculty, Vilnius, Lithuania; 33 Medical School, European University of Cyprus, Nicosia, Cyprus; 34 Tbilisi State University Faculty of Medicine, Tbilisi, Georgia; 35 Clinic of Infectious Chest Diseases, Dermatology and Allergology, Institute of Biomedical Sciences, Institute of Clinical Medicine, Vilnius University Faculty of Medicine, Vilnius, Lithuania; 36 Clinic of ENT and Eye Diseases, Institute of Clinical Medicine, Vilnius University Medical Faculty, Vilnius, Lithuania; 37 Danish Commitee for Health Education, Copenhagen East, Denmark; 38 Department of Prevention of Envinronmental Hazards and Allergology, Medical University of Warsaw, Warsaw, Poland; 39 Institute of Child and Adolescent Health at Arabkir Medical Centre, Yerevan State Medical University, Yerevan, Armenia; 40 Department of Immunology and Allergology, Medical Academy, Lithuanian University of Health Sciences, Kaunas, Lithuania; 41 European Health Futures Forum (EHFF), Dromahair, Ireland; 42 Clinic of Children’s Diseases, Medical Academy, Lithuanian University of Health Sciences, Kaunas, Lithuania; 43 Department of Rehabilitation, Physical and Sports Medicine, Institute of Health Sciences, Vilnius University Medical Faculty, Vilnius, Lithuania; 44 Department of Otorhinolaryngology, University Hospital Leuven, Leuven, Belgium; 45 Academic Medical Center, University of Amsterdam, Amsterdam, The Netherlands

**Keywords:** Air pollution, Asthma, Chronic respiratory diseases, Guidelines, Integrated care pathways, mHealth, Rhinitis, Vilnius Declaration

## Abstract

**Background:**

Over 1 billion people suffer from chronic respiratory diseases such as asthma, COPD, rhinitis and rhinosinusitis. They cause an enormous burden and are considered as major non-communicable diseases. Many patients are still uncontrolled and the cost of inaction is unacceptable. A meeting was held in Vilnius, Lithuania (March 23, 2018) under the patronage of the Ministry of Health and several scientific societies to propose multisectoral care pathways embedding guided self-management, mHealth and air pollution in selected chronic respiratory diseases (rhinitis, chronic rhinosinusitis, asthma and COPD). The meeting resulted in the Vilnius Declaration that was developed by the participants of the EU Summit on chronic respiratory diseases under the leadership of Euforea.

**Conclusion:**

The Vilnius Declaration represents an important step for the fight against air pollution in chronic respiratory diseases globally and has a clear strategic relevance with regard to the EU Health Strategy as it will bring added value to the existing public health knowledge.

## Background: chronic respiratory diseases are major chronic diseases across the life cycle

Over 1 billion people suffer from chronic respiratory diseases such as asthma, COPD, rhinitis and rhinosinusitis. Chronic respiratory diseases are major chronic diseases [1]. Asthma and allergic diseases occur along the life cycle from early childhood, affecting 30 million children and adults under 45 years of age in Europe. COPD has an estimated annual death rate of over 4 million people globally. The annual costs due to asthma (direct costs, indirect costs and loss of productivity) in the EU are estimated at 72 b€ and those due to COPD at 142 b€ [2]. Work productivity loss due to rhinitis is estimated at 30–50 b€ [3] and the disease has a major impact on sleep and anxiety [4]. Children with early onset asthma, active smoking, exposure to tobacco smoke and poverty are all risk factors of fixed airflow obstruction in adulthood [5–7].

The Polish Presidency of the EU Council (3051st Council Conclusions) made the prevention, early diagnosis and treatment of asthma and allergic diseases a priority to reduce health inequalities [8, 9]. The 3206th Cyprus Council Conclusions [10] recommended that the diagnosis and treatment of chronic diseases should be initiated as early as possible to improve active and healthy ageing (AHA). Several debates at the European Parliament recommended an early diagnosis and management of chronic respiratory diseases in children in order to promote AHA (Cyprus Presidency of the EU Council, 2012) [11], predictive medicine [12] and self-management strategies using mobile technology [13].

The Vilnius EU Summit on the prevention and management of chronic respiratory diseases (Vilnius, Lithuania, 23 March, 2018) followed these recommendations and attempted to provide a road map to prevent and control chronic respiratory diseases using integrated care pathways and the recent advances in mobile technology with a focus on air pollution.

## Protective and risk factors

Chronic respiratory diseases and other chronic diseases often share risk factors (e.g. tobacco, including e-cigarettes, allergen exposure, nutrition, indoor and outdoor air pollution and sedentary lifestyle) [1, 14, 15], all leading to sustained local and systemic inflammation [16, 17] as well as ageing. Tobacco smoking is the best identified risk factor for many chronic diseases including chronic respiratory diseases. Allergens are of importance in rhinitis and asthma. Indoor and outdoor air pollution is another important risk factor for chronic respiratory diseases of increasing importance [18–21]. Ecosystems are impacted by air pollution, particularly sulphur and nitrogen emissions, and by ground-level ozone: these factors affect their ability to function and grow and have adverse effects on flora and fauna as well as on allergic diseases [22]. Moreover, these risk factors can interact to worsen chronic respiratory diseases and induce exacerbations [23].

Pre-natal and early-life events have a major impact on the development of chronic diseases in adults [24, 25] and older people. A better understanding of these links will enable the implementation of effective novel prevention strategies to promote AHA. The developmental origin of ageing is on the EU political agenda [9, 10, 26, 27].

Chronic disease prevention and control could be considered sequentially before the disease has been identified (i) to prevent its onset (health promotion and primary prevention), and (ii) after its onset, to better control and prevent its short- and long-term consequences (secondary and tertiary prevention and control). It should also be noted that early impairment of lung function is a marker of early all-cause systemic morbidity and mortality [23–25].

Health promotion and prevention should start at conception and continue steadily across the life cycle for healthy lungs and active and healthy ageing. They have been laid down from a political perspective to contribute to the goals of the Europe 2020 strategy of healthy and active ageing [28], the Sustainable Development Goals for 2030 (SDG) and the WHO strategy on chronic diseases. The strategy was also promoted by the Polish EU priority [8, 9]. Multi-sectoral prevention, including policy change, regulation, and market intervention, is of the highest priority.

There is a need to find novel health promotion strategies in chronic respiratory diseases and to promote value creation in order to promote AHA. Particular attention is required for tobacco smoke prevention and for air pollution mitigation strategies. It should be noted that there is ample evidence of the effectiveness of legislation to restrict smoking and reduce pollution, and that Governments should therefore be pushed to implement the best environmental policy.

## Chronic respiratory diseases will be seriously impacted by climate change

Climate change is considered as a major threat for chronic respiratory diseases [29, 30]. Heat waves significantly increase morbidity and mortality in patients with chronic lung disease [31]. Climate change, mediated by greenhouse gases, causes adverse health effects to vulnerable chronic respiratory diseases patients including the elderly, children, and those in a distressed socioeconomic state [32]. Reducing greenhouse gas emission and improving air quality represent two global challenges [33].

## Poor adherence to medication: a major factor in chronic respiratory disease-related burden and death

Public health measures play an essential role in improving outcomes, but, for conditions where effective medications are available, such as for non-severe asthma, patients are supported in taking their medication effectively. One important global cause of low adherence is that medications are not accessible or affordable [14], and this issue must be addressed across Europe, especially in middle-income countries. If medications are accessible, adherence is relatively high. This is a known important factor in asthma deaths [15]. Unfortunately, the education programmes that have been proposed over the past decades have not increased adherence. Innovative effective educational programmes using mobile technology and guided self-management appear to be crucial for the empowerment of patients and will increase and support their adherence to medications. However, evidence-based real-life studies should be proposed to confirm the importance of mHealth and lead to change management We need to develop evidence-based strategies to change the management of CRDs [34]. School-based interventions are also of great importance. Ensuring that practical support strategies are in place also represents an essential strategy for improving outcomes. On-demand strategies may be of great interest for the improvement of adherence [35, 36].

## Prevention and treatment strategies at national or regional levels

Effective strategies are needed to reduce chronic respiratory disease burden. National programmes (Finnish, Czech, Portuguese Asthma or COPD Programmes) or national partnerships against chronic respiratory diseases (Italy, Netherlands, Lithuania, France, Romania, Belgium and Spain) can be cost-effective [37]. However, they are insufficiently implemented in many other EU countries, in particular in RIS countries [EIT Regional Innovation Scheme (EIT RIS)].

Integrated care pathways (ICPs) exist in some countries, such as the UK for COPD (QS10) (NICE) [38], France (HAS) and the Netherlands [39], but national ICPs for asthma or asthma and rhinitis comorbidity do not exist. Quality standards for asthma (QS25) have been published by NICE [40]. These are specific, concise statements that act as markers of high-quality and cost-effective patient care. Moreover, some initiatives are aimed at also incentivising good practice and improving implementation (i.e. remuneration based on performance indicators). In the UK, the Quality and Outcomes Framework (QOF) has 4 asthma-specific performance indicators which are explicitly linked to the subsequent remuneration of providers [41].

**Fig. 1:**
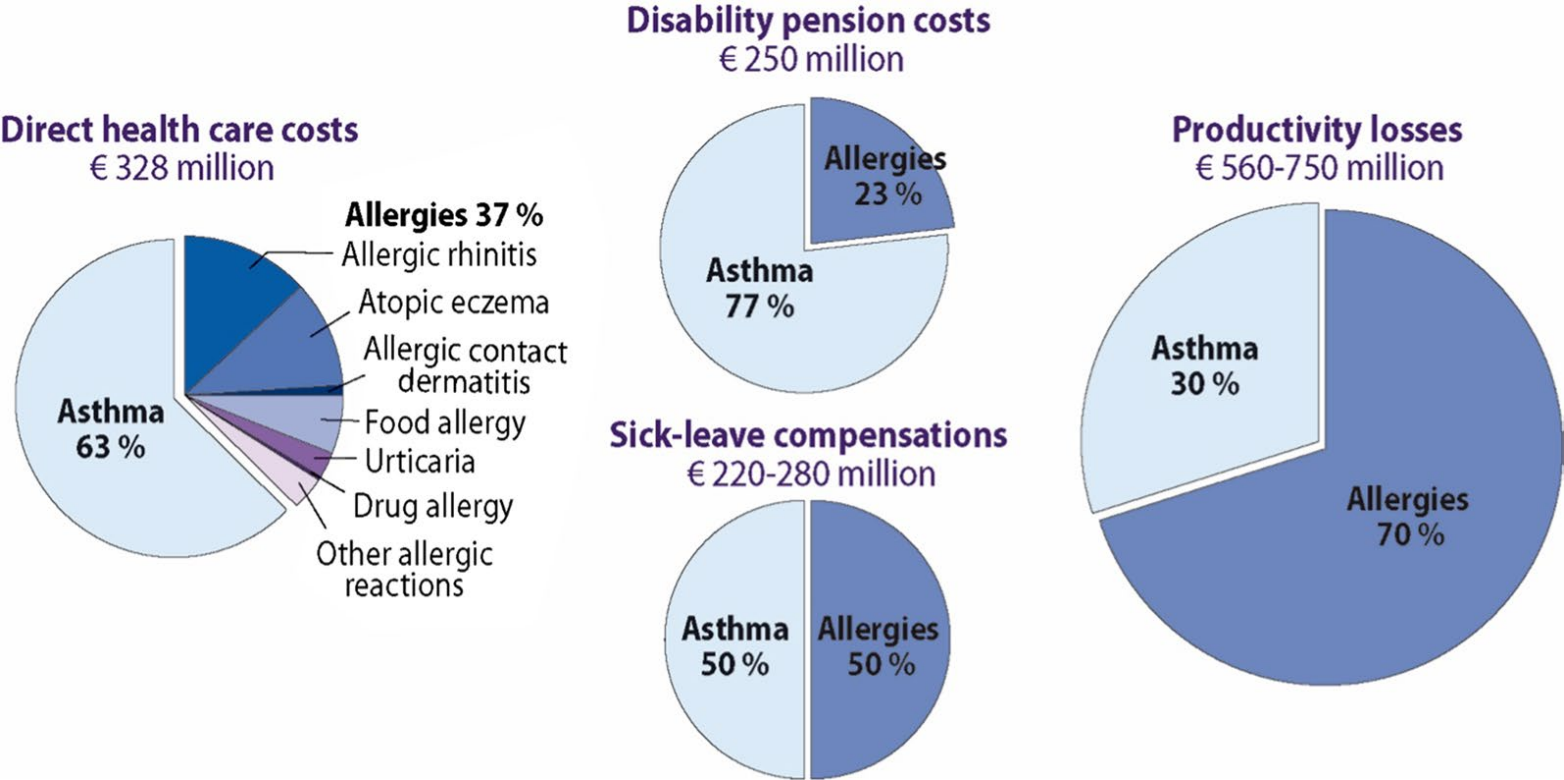
In 2013, direct and indirect asthma and allergy costs were €1.3–1.6 billion in Finland (population 5.5 million). From [50]

The Finnish Asthma 1994–2004 [42] and Allergy Programmes [43, 44] pioneered the field and have been found to be effective [45], demonstrating why public health problems need public health solutions. The programmes have been based on scientific innovations turned into practical actions. This has been possible in a country where the educational level of citizens is relatively high and where health care is efficiently organized and mainly funded by public money. Integration of the work of primary and secondary care physicians and nurses, pharmacists and caregivers has been the key for a better flow of information, education and patient care, while patient education and public awareness improved at the same time by organisations representing patients. However, the asthma programme has been successfully deployed in Europe [46] and developing countries [1, 37, 47]. The allergy programme has been deployed in Norway [48]. In asthma, improved detection of disease and recognizing asthma as an inflammatory disorder from the outset has led to early and effective management [49]. In the 2016 Pharmacy Barometer Survey, not more than 2.5% of Finnish asthma patients reported severe symptoms. In allergy, the *turning avoidance into tolerance* strategy, focusing on severe forms of disease and emphasizing health rather than mild problems, has encouraged a more rationale use of healthcare resources [3]. A nationwide cost analysis was also performed (Fig. 1) [50].

**Fig. 2:**
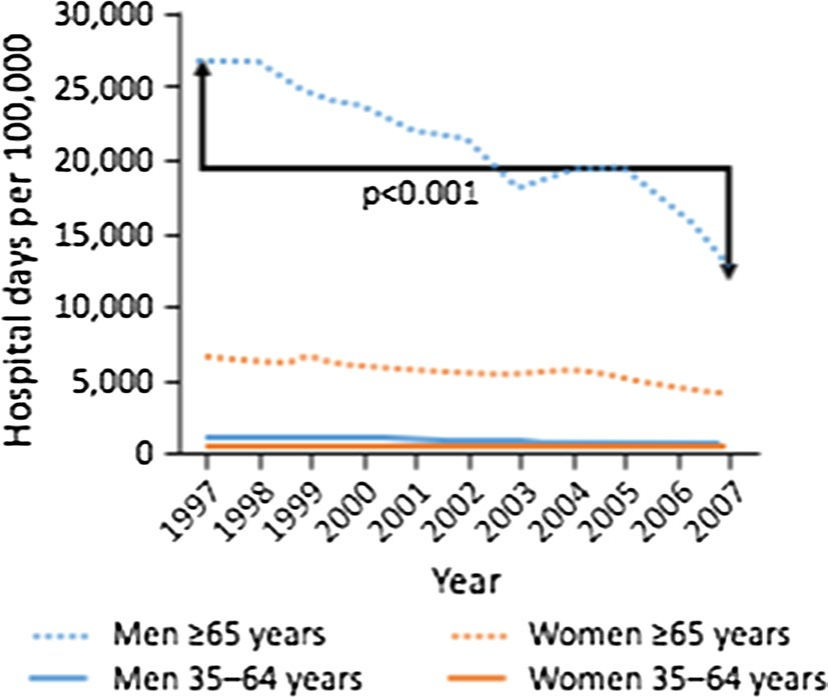
Hospital admissions were reduced in Finland after initiation of the 10-year COPD programme (from Kinnula et al. [51])

The Finish COPD programme has also been a success, the prevalence of smoking was reduced in both men and women, there was improved quality of diagnosis and reduction in hospitalizations (Fig. 2) [51].

The Finnish experience calls all European communities to take systematic and coordinated action to improve public health by lessening disability and costs caused by asthma and allergy. Inadequate care of these conditions seems to be a global problem delaying patient management and causing poor outcome [64]. Although many actions have already been taken in Europe [65], Lithuania and Baltic countries can speed up implementation and make it relevant to healthcare and society as a whole.

## Multisectoral care pathways embedding guided self-management, mHealth and air pollution in chronic respiratory diseases

ICPs are structured multidisciplinary care plans which detail essential steps in the care of patients [52]. They promote the translation of guidelines into local protocols and their subsequent application to clinical practice [53]. They empower patients and their carers (health and social). ICPs differ from practice guidelines as they are utilised by a multidisciplinary team and have a focus on the quality and coordination of care. Multisectoral care pathways for multimorbid chronic respiratory diseases are needed [54] and should incorporate self-management and aerobiology to allow precision medicine with endotype-driven treatment [55].

A large number of chronic respiratory disease patients do not consult physicians because they think their symptoms are ‘normal’ and/or trivial. However, chronic respiratory diseases including rhinitis and asthma negatively impact social life, school and work productivity [56]. There is a clear role for the pharmacist for AR management in practice, through a guided change management process.

An example of an ICP has been proposed for allergic rhinitis (AR). Many rhinitis patients use over-the-counter (OTC) drugs [57] and only a fraction have had a medical consultation. The vast majority of patients who visit GPs or specialists have moderate/severe rhinitis [58–62]. GRADE-based guidelines are available for AR and their recommendations are similar [63–65]. However, they are based on the assumption that patients regularly use their treatment and are not tested with real-life data. Unfortunately, adherence to treatment is very low and real-life studies do not necessarily accord with all recommendations [66]. New-generation guidelines embedding real life data are needed.

**Fig. 3:**
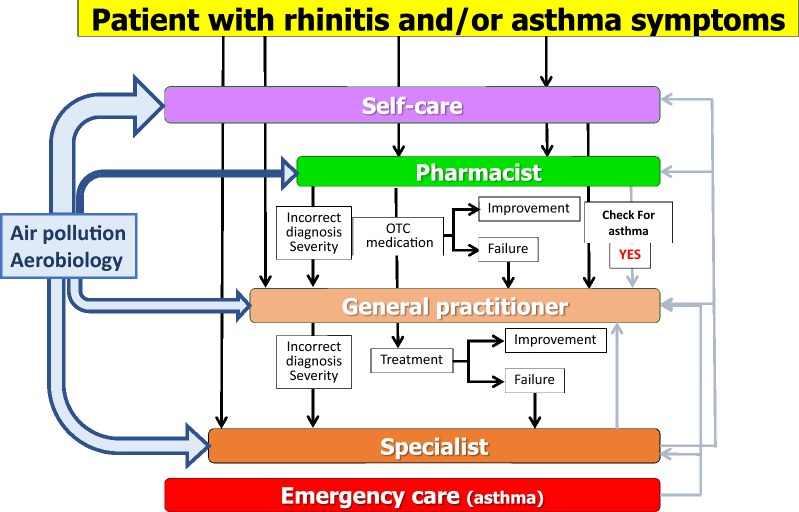
Integrated care pathways for rhinitis and asthma multimorbidity embedding aerobiology

Thus, ICPs should consider a multi-disciplinary approach as proposed by AIRWAYS ICPs (Fig. 3), using self-medication, shared decision making (SDM) and new-generation guidelines including messaging for increased adherence and information on aerobiology and air pollution.

### Self-care and shared decision making

Self-care is any necessary human regulatory function that is under individual control, deliberate and self-initiated. Chronic respiratory diseases require a long-term approach with emphasis on patient empowerment and appropriate support services stressing the importance of health literacy. In self-care, chronic disease patients make many day-to-day decisions. Self-management education complements traditional patient education. Self-care is learned, purposeful and continuous, otherwise it is dependent only on individuals’ specific circumstances, resources, beliefs, etc. Some important targets of self-care include the prevention of asthma and COPD exacerbations [67–69] but also the improvement of control of asthma, rhinitis or chronic rhinosinusitis [13, 70].

In shared decision making, both the patient and the health care professional contribute to the medical decision-making process, placing the patient at the centre of the decision [71]. For children, caregivers should contribute to the decision. Physicians explain treatments and alternatives to patients who can then choose the treatment option that best aligns with personal beliefs and goals and consider the benefits and risks [72]. In contrast to shared decision making, the traditional medical care system places physicians in a position of authority, with patients playing a passive role in care [73].

### mHealth including messaging

mHealth has evolved from eHealth, the use of information and communication technology (ICT) for health services and information transfer. According to the World Health Organization (WHO) [74], mHealth “has the potential to transform the face of health service delivery across the globe. A powerful combination of factors is driving this change. These include rapid advances in mobile technologies and applications, a rise in new opportunities for the integration of mobile health into existing mHealth services, and the continued growth in coverage of mobile cellular networks.” The potential applications and benefits of mHealth are extensive and expanding [75]. mHealth may be of great value in self-care and shared decision making.

The potential for mHealth in chronic respiratory diseases is enormous [76] but implementing ICT innovations may also have adverse consequences. It is therefore important to test applicability in each individual situation [77]. Apps may be used to better understand and improve adherence, in particular using messaging, as well as to enable shared decision making and improve self-management of chronic diseases [78]. Patients, who are the final users of these apps, must be included not only in the testing phase, but also in the design and creation [79].

## The Vilnius Declaration (VD)

### The VD is an essential step for the fight against air pollution in chronic respiratory disease globally

A first meeting on air pollution in chronic respiratory diseases and mHealth was held during the 11th GARD meeting in Brussels (November 10, 2017). It paved the way for the VD.

- POLLAR (Impact of Air POLLution on Asthma and Rhinitis) [80] is an EIT Health project that will propose strategies to predict and prevent air pollution peaks and chronic respiratory disease morbidity using mobile technology (Paris, January 2018).
- The Vilnius EU Summit (March 2018) prepared the VD and proposed a meeting at the UN with the following objectives: (i) to tackle the future risk of air pollution and climate change in chronic respiratory diseases across the life cycle, (ii) to propose practical primary and secondary prevention strategies to meet this unprecedented public health challenge and (iii) to combat inequities.
- The first ever WHO conference on air pollution and health was held in Geneva on October 30th–November 1st 2018, a clear sign that the problem is growing and that solutions are needed.
- The 12th GARD meeting in Helsinki (August 2018) refined the VD for the UN High-Level Conference.
- An ARIA-Masterclass (with Euforea) was the first teaching session on the subject and focussed on ICPs, self-management and shared decision making (Brussels, September 12 2018).
- A meeting (HLC) was organized at the UN Assembly (September 27, 2018), the results of which led to an action plan for implementing recommendations.
- A consensus meeting was organized (POLLAR, Paris, December 2018) to improve care pathway design in order to enhance patient participation, health literacy and self-care through technology-assisted ‘patient activation’ using rhinitis and asthma multimorbidity as a model of non-communicable disease. Pharmacy guidelines were included in this meeting.
- With the results of POLLAR, a consensus meeting was organized (POLLAR, Paris, December 2019) to embed aerobiology and air pollution in ICPs.

### The VD

The Vilnius Declaration was developed by the participants of the EU Summit on chronic respiratory diseases (Vilnius, March 23, 2018) under the leadership of Euforea (“Appendix”).

### The expected impact of the VD

The VD has a clear strategic relevance with regard to the EU Health Strategy as it will bring added value to the existing public health knowledge:

- To propose a common framework for integrated care pathways in chronic respiratory diseases (and their co-morbidities) for the entire EU which can be expanded to Eastern European and other regions and which will allow comparability and trans-national initiatives;
- To help risk stratification in chronic respiratory disease patients with a common strategy;
- To develop cost-effective policies, in particular strengthening those on smoking, including e-cigarettes, and environmental exposure;
- To propose a common simulation tool for the entire EU to assist physicians in the segmentation of patients and choice of pathways;
- To develop the interoperability of mHealth systems in Europe, including transborder interoperability in Eastern European regions (border between EU member states and associate EU countries);
- To have a significant impact on the health of citizens in the short term (reduction of morbidity, improvement of education in children and of work in adults) and in the long term (healthy ageing).

## Conclusions

The Vilnius Declaration represents an important step for the fight against air pollution in chronic respiratory diseases globally and has a clear strategic relevance with regard to the EU Health Strategy as it will bring added value to the existing public health knowledge.

## Appendix: the Vilnius Declaration

### Introduction

1. The European Forum for Research and Education in Allergy and Airway Diseases (Euforea) has developed the Vilnius Declaration as a statement to improve the guided self-management of chronic respiratory diseases using mHealth and giving special consideration to the impact of air pollution and climate change.
2. The Declaration was elaborated and approved during the EU Summit on chronic respiratory diseases in Vilnius, Lithuania, on 23 March 2018, and addressed to health care personnel, policy makers and, most importantly, to patients suffering from chronic respiratory diseases.
3. Over 1 billion people suffer from chronic respiratory diseases such as asthma, Chronic Obstructive Pulmonary Disease (COPD), rhinitis and rhinosinusitis. Chronic respiratory diseases are major chronic diseases.
4. It is the duty of the medical community and health care systems to provide patients with tools to assist them with the most appropriate treatment and self-management of their condition.

### Chronic respiratory diseases are major chronic diseases across the life cycle and are affected by environmental risk factors

5. Chronic respiratory diseases and other chronic diseases often share environmental risk factors (e.g. tobacco use, including e-cigarettes, nutrition, indoor and outdoor air pollution and sedentary lifestyle), leading to sustained local and systemic inflammation as well as impaired ageing.
6. Asthma and allergic diseases occur along the life cycle from early childhood, affecting 30 million children and adults under 45 years of age in Europe and more than 600 million people globally. COPD is responsible for an estimated 3 million deaths globally.
7. Pre-natal and early-life events have a major impact on the development of chronic diseases in adults and older people. Early impairment of lung function is a marker of early all-cause systemic morbidity and mortality.
8. Smoking is the most important risk factor for chronic respiratory diseases. Indoor and outdoor air pollution are other important risk factors of increasing importance. Allergens are of importance in rhinitis and asthma. Moreover, these risk factors can interact to worsen chronic respiratory diseases and induce exacerbations.

#### Prevention, control and self-management of chronic respiratory diseases

9. Chronic disease prevention and control could be considered sequentially before the disease has been identified (i) to prevent its onset (health promotion and primary prevention), and (ii) after its onset, to better control and prevent its short- and long-term consequences (secondary and tertiary prevention and control).
10. Tobacco smoking is the best identified risk factor for many chronic diseases including chronic respiratory diseases.
11. Health promotion and prevention should start at conception and be steadily continued across the life cycle for healthy lungs and active and healthy ageing.
12. A large number of chronic respiratory disease patients do not consult physicians because they think their symptoms are trivial. However, chronic respiratory diseases, including rhinitis and asthma, negatively impact social life, school and work productivity.
13. Many rhinitis patients use over-the-counter (OTC) drugs and only a fraction have had a medical consultation. The vast majority of patients who visit general practitioners or specialists have moderate/severe rhinitis.
14. Thus, Integrated Care Pathways should consider a multi-disciplinary approach including physicians, pharmacists and other health care personnel as proposed by AIRWAYS ICPs. Self-medication and shared decision making should be used as well as new-generation guidelines including messaging for increased adherence and information on aerobiology and air pollution.
15. Chronic respiratory diseases require a long-term approach with emphasis on patient empowerment and appropriate support services stressing the importance of health literacy.
16. Self-care is any necessary human regulatory function which is under individual control, deliberate and self-initiated.
17. In shared decision making, both the patient and the physician contribute to the medical decision-making process, placing the patient at the centre of the decision. Physicians explain treatments and alternatives to patients who can then choose the treatment option that best aligns with personal beliefs and goals and consider the benefits and risks.
18. The potential for mHealth in chronic respiratory diseases is enormous but implementing Information and Communication Technology (ICT) innovations may also have adverse consequences. It is therefore important to test applicability in each individual situation.
19. Mobile Applications (Apps) may be used to better understand and improve adherence, in particular using messaging.

### Health system strengthening and Sustainable Development Goals (SDG)

The Vilnius Declaration aims to contribute to the following actions:

20. To propose a common framework for integrated care pathways in chronic respiratory diseases (and their co-morbidities) for the entire EU which can be expanded to Eastern European and other regions and which will allow comparability and trans-national initiatives.
21. To extend the Finnish experience of population management models of long-term state chronic respiratory disease control programmes, especially in low to middle income countries.
22. To help risk stratification in chronic respiratory disease patients with a common strategy.
23. To develop cost-effective policies, in particular strengthening those on the reduction of smoking, including e-cigarettes, and environmental exposure.
24. To propose a common simulation tool for the entire EU to assist physicians in the segmentation of patients and choice of pathways.
25. To develop interoperability of mHealth systems in Europe, including transborder interoperability in Eastern European regions (border between EU member states and associate EU countries).
26. To have a significant implication on the health of citizens in the short term (reduction of morbidity, improvement of education in children and of work in adults) and in the long term (healthy ageing).
27. To achieve the goals of the Europe 2020 strategy of healthy and active ageing and of the UN Sustainable Development Goals to be reached in 2030.

**Table:**

European Forum for Research and Education in Allergy and Airway Diseases (Euforea) Ministry of Health of Lithuania Ministry of Health of Ukraine Ministry of Labour, Health and Social Affairs of Georgia Ministry of Health, Labour and Social Protection of Republic of Moldova Allergic Rhinitis and its Impact on Asthma (ARIA) Committee of Health of Lithuanian Seimas (Parliament) European Academy of Allergy and Clinical Immunology (EAACI) European Academy of Paediatrics (EAP/UEMS-SP) European Association of Allergy and Asthma Patients’ Associations (EFA) European Respiratory Society (ERS) European Rhinology Society (ERS) Lithuanian Academy of Sciences Reference Site Network of the European Innovation Partnership on Active and Healthy Ageing (RSCN-EIP on AHA) Vilnius University Medical Faculty Global Alliance Against Chronic Respiratory Diseases (GARD, WHO Alliance) World Health Organization (WHO) Lithuanian Office	

## Abbreviations

AHA: active and healthy ageing
AR: allergic rhinitis
ARIA: Allergic Rhinitis and its Impact on Asthma
COPD: chronic obstructive pulmonary disease
EIT: European Institute for Innovation and Technology
EU: European Union
Euforea: European Forum for Research and Education in Allergy
GARD: Global Alliance Against Chronic Respiratory Diseases
GRADE: Grading of Recommendations Assessment, Development and Evaluation
ICP: integrated care pathway
ICT: information and communication technology
NICE: National Insitute for Health and Care Excellence
OTC: over the counter
POLLAR: Impact of Air POLLution on Asthma and Rhinitis
SDG: Sustainable Development Goals
VD: Vilnius Declaration
WHO: World Health Organization
