# Correction to: Vilnius Declaration on chronic respiratory diseases: multisectoral care pathways embedding guided self-management, mHealth and air pollution in chronic respiratory diseases

**DOI:** 10.1186/s13601-020-00357-4

**Published:** 2020-11-17

**Authors:** Arunas Valiulis, J. Bousquet, A. Veryga, U. Suprun, D. Sergeenko, S. Cebotari, D. Borelli, S. Pietikainen, J. Banys, I. Agache, N. E. Billo, A. Bush, I. Chkhaidze, L. Dubey, W. J. Fokkens, J. Grigg, T. Haahtela, K. Julge, O. Katilov, N. Khaltaev, M. Odemyr, S. Palkonen, R. Savli, A. Utkus, V. Vilc, T. Alasevicius, A. Bedbrook, M. Bewick, J. Chorostowska-Wynimko, E. Danila, A. Hadjipanayis, R. Karseladze, V. Kvedariene, E. Lesinskas, L. Münter, B. Samolinski, S. Sargsyan, B. Sitkauskiene, D. Somekh, L. Vaideliene, Algirdas Valiulis, P. W. Hellings

**Affiliations:** 1 Department of Public Health, Institute of Health Sciences, and Clinic of Children’s Diseases, Institute of Clinical Medicine, Vilnius University Faculty of Medicine, Vilnius, Lithuania; 2 European Academy of Paediatrics (EAP/UEMS-SP), Brussels, Belgium; 3 MACVIA-France, Fondation Partenariale FMC VIA-LR, CHU Montpellier, 371 Avenue du Doyen Gaston Giraud, 34295 Montpellier Cedex 5, France; 4 INSERM U 1168, VIMA: Ageing and Chronic Diseases Epidemiological and Public Health Approaches, Villejuif, France; 5 UMR‑S 1168, Université Versailles St-Quentin-en-Yvelines, Montigny le Bretonneux, France; 6 Euforea, Brussels, Belgium; 7 Charité, Berlin, Germany; 8 Minister of Health, Vilnius, Lithuania; 9 Minister of Health, Kiev, Ukraine; 10 Minister of Labour, Health and Social Affairs, Tbilisi, Georgia; 11 Minister of Health, Labour and Social Protection, Chișinău, Moldova; 12 European Parliament, Perugia, Italy; 13 European Parliament, Helsinki, Finland; 14 Lithuianian Academy of Sciences, Vilnius, Lithuania; 15 Faculty of Medicine, Transylvania University, Brasov, Romania; 16 Global Alliance Against Chronic Respiratory Diseases (GARD), Joensuu, Finland; 17 Imperial College and Royal Brompton Hospital, London, UK; 18 Department of Pediatrics, and Iashvili Central Children’s Hospital, Tbilisi State Medical University, Tbilisi, Georgia; 19 Faculty of Postgraduate Education, Lviv National Medical University by Danylo Halytsky, Lviv, Ukraine; 20 Department of Otorhinolaryngology, Amsterdam University Medical Centres, AMC, Amsterdam, The Netherlands; 21 Centre for Genomics and Child Health, Blizard Institute, Queen Mary University of London, London, UK; 22 Skin and Allergy Hospital, Helsinki University Hospital and University of Helsinki, Helsinki, Finland; 23 Children’s Clinic, Tartu University Institute of Clinical Medicine, Tartu, Estonia; 24 Vinnytsa National Medical University by Mykola Pyrogov, Vinnytsa, Ukraine; 25 Global Alliance Against Chronic Respiratory Diseases (GARD-WHO), Geneva, Switzerland; 26 European Federation of Allergy and Airways Diseases Patients’ Associations (EFA), Brussels, Belgium; 27 Department of Human and Medical Genetics, Institute of Biomedical Sciences, Vilnius University Faculty of Medicine, Vilnius, Lithuania; 28 Association of Medical Schools in Europe, Berlin, Germany; 29 State Institute of Phtysiopulmonology by Chiril Draganiuk, Chisinau, Moldova; 30 iQ4U Consultants Ltd, London, UK; 31 Department of Genetics and Clinical Immunology, National Institute of Tuberculosis and Lung Diseases, Warsaw, Poland; 32 Clinic of Chest Diseases, Immunology and Allergology, Centre of Pulmonology and Allergology, Institute of Clinical Medicine, Vilnius University Medical Faculty, Vilnius, Lithuania; 33 Medical School, European University of Cyprus, Nicosia, Cyprus; 34 Tbilisi State University Faculty of Medicine, Tbilisi, Georgia; 35 Clinic of Infectious Chest Diseases, Dermatology and Allergology, Institute of Biomedical Sciences, Institute of Clinical Medicine, Vilnius University Faculty of Medicine, Vilnius, Lithuania; 36 Clinic of ENT and Eye Diseases, Institute of Clinical Medicine, Vilnius University Medical Faculty, Vilnius, Lithuania; 37 Danish Commitee for Health Education, Copenhagen East, Denmark; 38 Department of Prevention of Envinronmental Hazards and Allergology, Medical University of Warsaw, Warsaw, Poland; 39 Institute of Child and Adolescent Health at Arabkir Medical Centre, Yerevan State Medical University, Yerevan, Armenia; 40 Department of Immunology and Allergology, Medical Academy, Lithuanian University of Health Sciences, Kaunas, Lithuania; 41 European Health Futures Forum (EHFF), Dromahair, Ireland; 42 Clinic of Children’s Diseases, Medical Academy, Lithuanian University of Health Sciences, Kaunas, Lithuania; 43 Department of Rehabilitation, Physical and Sports Medicine, Institute of Health Sciences, Vilnius University Medical Faculty, Vilnius, Lithuania; 44 Department of Otorhinolaryngology, University Hospital Leuven, Leuven, Belgium; 45 Academic Medical Center, University of Amsterdam, Amsterdam, The Netherlands

## Correction to: Clin Transl Allergy (2019) 9:7. https://doi.org/10.1186/s13601-019-0242-2

Following publication of the original article [1], in issue was identified with distinguishing the 1st from the 41st authors, both named ‘A. Valiulis’. This caused errors with correctly linking these authors on third party indexing websites.

The author names for the 1st and 41st author have been expanded to Arunas Valiulis and Algirdas Valiulis in the author list of this Correction article, in order to circumvent the linking errors.

In addition, the presentation of affiliation 1 has been corrected. Affiliation 1 was originally presented as ‘Department of Public Health, Clinic of Children’s Diseases, and Institute of Health Sciences, Vilnius University Institute of Clinical Medicine, Vilnius, Lithuania.’ This affiliation is corrected in the affiliation list of this Correction article to ‘Department of Public Health, Institute of Health Sciences, and Clinic of Children’s Diseases, Institute of Clinical Medicine, Vilnius University Faculty of Medicine, Vilnius, Lithuania’.
